# Novel triple-reassortant influenza viruses in pigs, Guangxi, China

**DOI:** 10.1038/s41426-018-0088-z

**Published:** 2018-05-16

**Authors:** Ping He, Guojun Wang, Yanning Mo, Qingxiong Yu, Xiong Xiao, Wenjuan Yang, Weifeng Zhao, Xuan Guo, Qiong Chen, Jianqiao He, Mingli Liang, Jian Zhu, Yangbao Ding, Zuzhang Wei, Kang Ouyang, Fang Liu, Hui Jian, Weijian Huang, Adolfo García-Sastre, Ying Chen

**Affiliations:** 1 College of Animal Science and Technology, Guangxi University, No.100 Daxue Road, Nanning, 530004 China; 2 Department of Microbiology, Icahn School of Medicine at Mount Sinai, New York, NY 10029 USA; 3 Guangxi State Farms Yongxin Jinguang Animal Husbandry Group Co., Ltd, Nanning, 530042 China; 4 Gentry Biotechnology Laboratories, Guangdong, 528437 China; 5 Global Health and Emerging Pathogens Institute, Icahn School of Medicine at Mount Sinai, New York, NY 10029 USA; 6 Department of Medicine, Division of Infectious Diseases, Icahn School of Medicine at Mount Sinai, New York, NY 10029 USA

## Abstract

Considered a “mixing vessel” for influenza viruses, pigs can give rise to new influenza virus reassortants that can threaten humans. During our surveillance of pigs in Guangxi, China from 2013 to 2015, we isolated 11 H1N1 and three H3N2 influenza A viruses of swine origin (IAVs-S). Out of the 14, we detected ten novel triple-reassortant viruses, which contained surface genes (hemagglutinin and neuraminidase) from Eurasian avian-like (EA) H1N1 or seasonal human-like H3N2, matrix (M) genes from H1N1/2009 pandemic or EA H1N1, nonstructural (NS) genes from classical swine, and the remaining genes from H1N1/2009 pandemic. Mouse studies indicate that these IAVs-S replicate efficiently without prior adaptation, with some isolates demonstrating lethality. Notably, the reassortant EA H1N1 viruses with EA-like M gene have been reported in human infections. Further investigations will help to assess the potential risk of these novel triple-reassortant viruses to humans.

## Introduction

The segmented nature of the genome of influenza viruses is responsible for its ability to reassort its eight RNA genes when cells are co-infected with two different influenza virus strains^1^. Influenza A virus of swine origin (IAV-S) circulating in pigs have been known to occasionally reassort with avian and human influenza viruses to generate novel genotypes and establish new swine influenza virus lineages. Importantly, these new lineages might represent influenza pandemic threats for humans, as highlighted by the last 2009 H1N1 influenza virus pandemic^2^. Despite several introductions of avian influenza virus genes into swine strains by reassortment, only viruses from H1, H2, and H3 subtypes out of the 16 possible avian influenza virus subtypes have been known to circulate in pigs in the last century, mirroring the same subtypes that have been known to circulate in humans.

At present, there are H1N1, H1N2, and H3N2 major viral subtypes circulating among pigs worldwide. Among the swine H1N1 influenza lineages, the classical H1N1 swine (CS) lineage is related to the H1N1 virus that caused both the 1918 and 2009 human pandemics^3,4^. The Eurasian avian-like (EA) H1N1 viruses have been prevalent in European pigs since 1979^5^ and had contributed its neuraminidase (NA) and matrix (M) segments to the H1N1/2009 pandemic virus^6^. In 1997–1998, double or triple-reassortant H3N2 strains emerged containing genes from classical swine H1N1, seasonal human, and avian viruses, and they became prevalent in swine in North America^7–9^. Following the outbreak of H1N1/2009 pandemic in humans, reassortant viruses derived from H1N1/2009 pandemic viruses and enzootic IAVs-S have been continuously detected from pigs in many regions globally^10–19^. Furthermore, sporadic human infection with EA H1N1 viruses and with EA-H1N1/2009 pandemic reassortant viruses has been reported^20–23^. Also, human infections with the swine-derived H3N2 variant (H3N2v) have been reported and they are associated with a IAV-S reassortant H3N2 strain that contains the M segment of the H1N1/2009 pandemic virus^24^, emphasizing the important role of pigs in the generation of reassortant influenza viruses with the potential to infect humans. Importantly, humans lack herd immunity to EA H1N1 viruses^11,25^, individuals aged ≤14 years show broad susceptibility against H3N2v viruses, and the seasonal human vaccine does not induce neutralizing antibodies against these IAVs-S^26^. The insufficient immunity in humans against H1 and H3 IAVs-S raises concerns over whether they could be the source of the next influenza pandemic.

Southern China has diverse IAV-S ecosystem, where H1N1, H1N2, and H3N2 subtypes and different lineages IAV-S are co-circulating in pigs^6,11,27^; however, IAV-S surveillance in this region of the world is still very limited. In this study, we performed surveillance in pigs in Guangxi. We found that novel triple-reassortant EA H1N1 and human-like (HL) H3N2 reassortants, which carried H1N1/2009 pandemic internal genes (PB2, PB1, PA, and NP) and CS H1N1 NS genes have been circulated in pigs for some time and they are predominant in the pig population in Guangxi. This is the first evidence that such novel IAV-S reassortants have been established in pigs in Guangxi, China. These viruses demonstrated variable levels of virulence in mice with some isolates being lethal in this animal model. We suggest that these novel IAV-S lineages should be closely monitored for their potential to cause human infections.

## Materials and methods

### Surveillance of IAV-S

From 2013 to 2015, disease outbreaks with severe respiratory signs, including fever, coughing, sneezing, nasal discharge, and low appetite occurred in ten pig farms in Guangxi. A total of 600 nasal swabs and 135 lung tissues with or without any respiratory signs were collected from farms or slaughterhouses in Nanning, Qinzhou, Chongzuo, Laibin, Liuzhou, Bobai, Guigang, Dongxing, and Baise of Guangxi (Table 1).

**Table 1:** Details of samples collected for SIVs testing in Guangxi

City	Farm	Strains abbreviation	Sampling site	Time	Clinical signs^a^
Nanning	1#	NN1994	Farm(500 sows)	2013.09	Difficulty breathing; PRRSV positive
	2#	NNLX	Farm (900 sows)	2014.10	Fever, coughing, sneezing, nasal discharge
	3#	NNXD	Farm(600 sows)	2013.01	Difficulty breathing; PRRSV and PCV positive
		NNXD2023	Farm(600 sows)	2013.09	Coughing, sneezing, nasal discharge
	4#	JGB4	Farm(1000 sows)	2013.09	Coughing and sneezing
		JG1	Farm(1000 sows)	2014.03	Coughing and sneezing
Chongzuo	5#	CZ7	Farm(1000 sows)	2014.10	Fever, coughing, sneezing
Qingzhou	6#	QZ5	Farm(3000 sows)	2014.08	Fever, coughing, nasal discharge
Laibing	7#	LB9	Farm(800 sows)	2014.10	Fever, coughing, sneezing
Liuzhou	8#	LZA3	Farm(500 sows)	2015.06	Coughing and sneezing
Bobai	9#	BB2	Farm(1000 sows)	2014.09	Coughing and sneezing
Guigang	10#	GG6	Farm(800 sows)	2013.05	Coughing and sneezing
Dongxing	N/A	DX24	Abattoirs	2013.12	N/A
Baise	N/A	BS30	Abattoirs	2014.11	N/A

*N/A* not available, *PCV* porcine circovirus

^a^PRRSV represents porcine reproductive and respiratory syndrome virus

### Virus isolation and DNA Sequencing

As previously described^28^, RNA was directly extracted from nasal swabs or lung tissues. Reverse transcription was carried out under standard conditions with universal 12 primer. One pair of universal M gene primers was used to amplify M gene. M gene positive samples were inoculated onto monolayers of Madin–Darby canine kidney (MDCK) cells maintained in DMEM containing 1 μg/mL trypsin for 48 h at 37 °C. After three serial passages, virus isolates were identified by RT-PCR and the whole genome was amplified by eight pairs of primers and sequenced.

### Phylogenetic analysis

Individual gene segments were aligned and analyzed with the Seqman and Megalign program (DNASTAR, Madison, USA). Phylogenetic trees were generated by applying the method of the Clustal IW alignment algorithm from MEGA 7.0 software (http://www.megasoftware.net/), and bootstrap values of 1000 were used.

### Serological assays

A total of 1170 swine serum samples were collected from different cities of Guangxi and treated with receptor-destroying enzyme (Sigma, USA) for 18 h at 37 °C, followed by heat inactivation at 56 °C for 30 min before being tested for the presence of hemagglutination-inhibition (HI) antibodies with 1% (V/V) guinea pig erythrocytes^29^. HI assays were performed on all treated serum samples with two contemporary viruses, EA H1N1 virus (A/swine/Guangxi/BB2/2013) and HL H3N2 virus (A/swine/Guangxi/JGB4/2013). HI antibody titers ≥20 were determined as serological positive.

### Replication and pathogenicity in mice

Groups of nine 6-week-old female BALB/c mice were anesthetized with CO_2_ and inoculated intranasally (i.n.) with 5 × 10^4^ TCID_50_ of selected influenza viruses in a volume of 50 µL. Mock-infected mice were inoculated i.n. with 50 µL phosphate-buffered saline. Three mice were killed on days 3 and 5 post infection (p.i.), respectively. Organs (spleen, kidney, intestine, brain, and lung) were collected to assess for viral replication in MDCK cells. The remaining mice were monitored for clinical signs, body weight, and survival for 14 days. Mice were killed when the body weight loss was above 25% of their pre-challenge weight.

## Results

### Virus isolation and identification

A total of 735 nasal swabs or lung tissue samples were collected from pigs on farms and in slaughterhouses in Guangxi province of southern China from 2013 to 2015, containing 55 positive samples identified by RT-PCR. Fourteen strains were successfully isolated by serial passages on MDCK cells, including 11 H1N1 and three H3N2 IAVs-S. These results indicate that different subtypes of IAVs-S co-circulating in Guangxi. The nucleotide sequences of the 14 IAVs-S determined in this study will be deposited in the GenBank database under numbers KM061010 to KM061025 and MF927789 to MF927884.

### Phylogenetic analysis

**Fig. 1:**
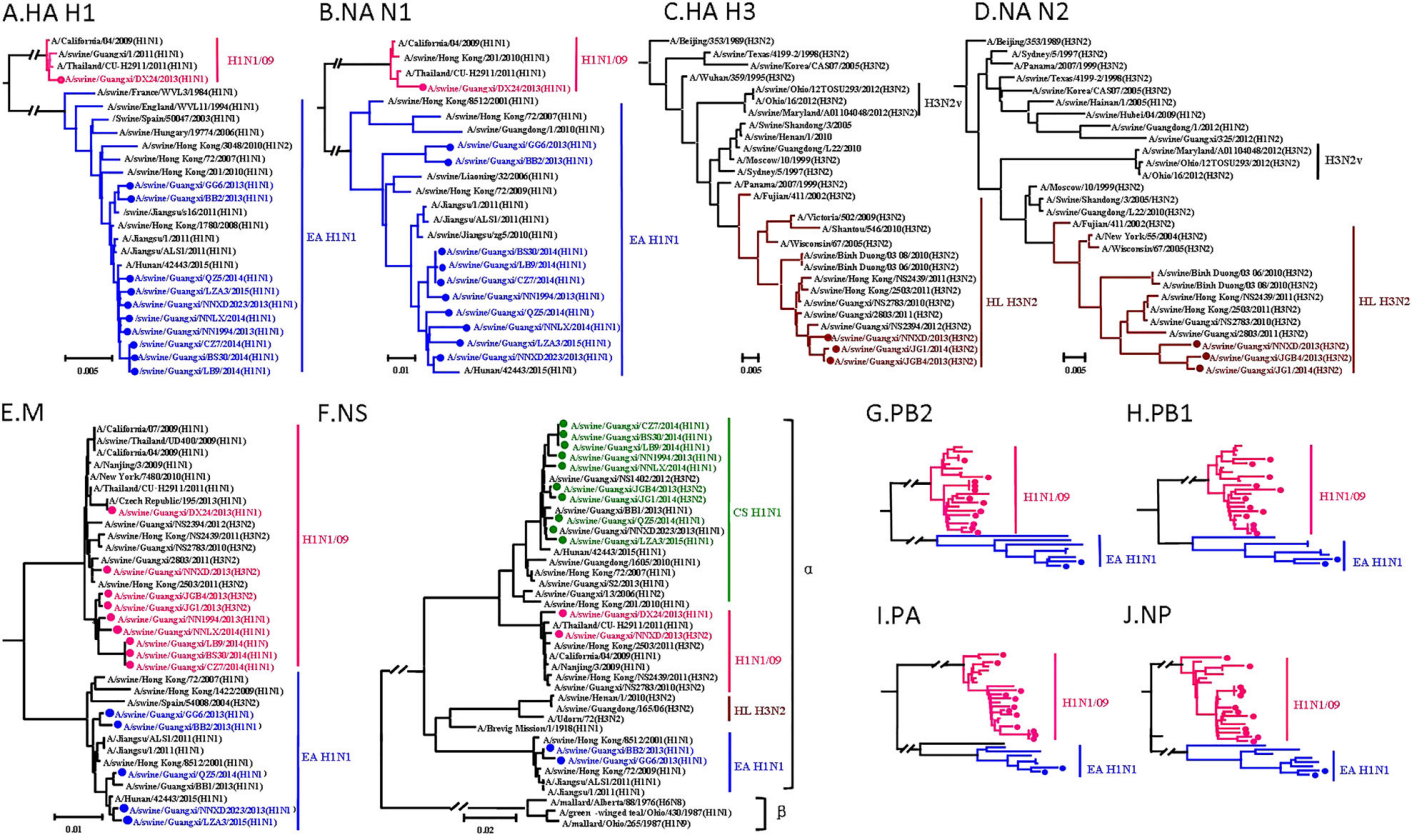
Phylogenetic trees of the HA H1 (**a**), NA N1 (**b**), HA H3 (**c**), NA N2 (**d**), M (**e**), NS (**f**), PB2 (**g**), PB1 (**h**), PA (**i**), and NP (**j**) genes of the H1N1 and H3N2 influenza lineages. The unrooted trees were generated with the MEGA 7.0 program by using neighbor-joining analysis and reliability of the tree was assessed by bootstrap analysis with 1000 replications. Neighbor-joining bootstrap values ≥70 are shown at the major branches of the trees. The 12 trees were rooted to A/Brevig_Mission/1/18(H1N1). Viruses shown in black were downloaded from available databases. The isolates in our study were marked in different color, consistent with Fig. 2

To determine the genetic characteristics of these viruses, all eight gene segments of the viruses grown in MDCKs were sequenced and phylogenetic analyses were performed. Phylogenetic analysis of the hemagglutinin (HA) reveals that the 11 H1 HA genes are separated into EA-like H1N1 lineage and H1N1/2009 pandemic lineage (Fig. 1a). All ten H1 HA genes clustered into the EA H1N1 lineage share 95.9–100% identity at the nucleotide level, whereas the interlineage homology is <74%. All the three H3 HA genes share over 98.6% of identity and are clustered into HL H3N2 lineage (Fig. 1c). Similar to the HA genes, ten N1 NA genes and one N1 NA gene are clustered into the EA H1N1 lineage and H1N1/2009 pandemic lineage, respectively (Fig. 1b). The N1 intralineage virus homology is over 94.7%, while the homology between the lineages is <89.4%. Three N2 NA genes are clustered into HL H3N2 lineage, which share over 98.7% of homology within the lineage (Fig. 1d).

The PB2, PB1, PA, NP, and M genes share 83–100%, 84.2–100%, 81.2–100%, 82.4–100%, and 92.5–100% identity, respectively, at the nucleotide level. The PB2, PB1, PA, and NP genes show the same clustering pattern. Specifically, 12 out of 14 viruses are clustered with the H1N1/2009 pandemic lineages, while the remaining two viruses belong to the EA H1N1 lineage (Fig. 1g–j and Figure S1). The intralineage homologies are above 97.1%, 96.7%, 95.3%, and 96.9%, respectively. However, the interlineage homologies are <84%, 84.8%, 84.7%, and 83.5%, respectively. The M gene is also clustered into two lineages: nine H1N1/2009 pandemic and five EA H1N1 (Fig. 1e). The homology of the M genes within lineages is over 96.2%, and the homology of the M genes is <95.6% between the lineages. Remarkably, the NS gene shows distinct diversity, forming three different lineages: EA H1N1 (2), H1N1/2009 pandemic (2), and CS H1N1 (10) (Fig. 1f). The NS intralineage virus homology is over 97.4%, while the interlineage homology is <91.4%. All of these NS genes belong to the alpha lineage nomenclature of NS gene, as shown in Fig. 1f.

**Fig. 2:**
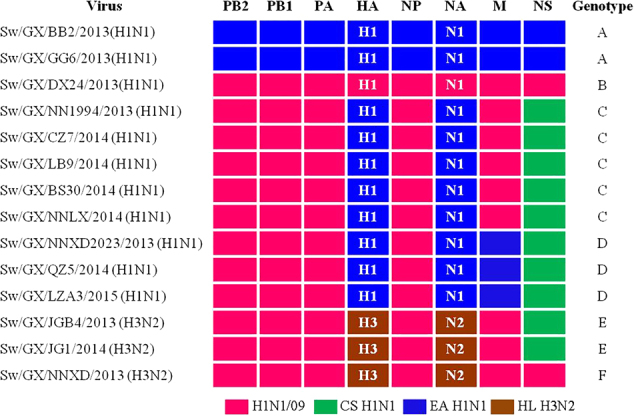
Genotypes of H1N1 and H3N2 IAVs-S from Guangxi during 2013 to 2015. Origin of each gene segment is colored for representing the different lineages

Based on phylogenetic analyses of the gene segments, the viruses isolated and grown in MDCKs can be divided into six distinct genotypes (A, B, C, D, E, and F) (Fig. 2). The IAVs-S in genotypes A and B are prototypical EA H1N1 and H1N1/2009 pandemic, respectively. Notably, the IAVs-S in genotype C are novel triple H1N1 reassortants containing the HA and NA genes from EA H1N1, the NS gene from CS H1N1 and the rest of the gene segments are from H1N1/2009 pandemic. The genotype D IAVs-S is similar to the genotype C, except for the M gene, which is derived from EA H1N1. Genotype D IAVs-S were first reported in Tianjin, China in 2013^30^ and caused human infection in Hunan, China in 2015^22^, this genotype was detected continuously from 2013 to 2015 in Guangxi in our study. The three H3N2 viruses in genotypes E and F have HA and NA genes derived from swine HL H3N2 viruses. It should be noted that two viruses in genotype E are also novel triple reassortants containing five H1N1/2009 pandemic-origin internal genes and the NS gene derived from CS, similar to H1N1 viruses in genotype C. One virus in genotype F possessed six internal genes from H1N1/2009 pandemic viruses, which was soon detected after the spillovers of H1N1/2009 pandemic from human to pigs^12^. Our data demonstrate that there are EA H1N1 and HL H3N2 IAVs-S which have reassorted to acquire the CS H1N1 NS gene and H1N1/2009 pandemic internal genes. The similar pattern of internal gene reassortment acquired by multiple H1N1 and H3N2 IAVs-S from different times and locations in Guangxi suggest that H1N1/2009 pandemic ribonucleoprotein complex genes (PB2, PB1, PA, and NP genes) and CS H1N1 NS gene constellation is selectively advantageous and compatible with different surface genes. Importantly, IAV-S has the potential to cause epidemics in humans. The triple reassortant, A/swine/Guangxi/NNXD2023/2013(H1N1) (Sw/GX/NNXD2023/2013) in genotype D, shared 98.6–99.3% similarity at the nucleotide level with the recent human isolate A/Hunan/42443/2015 (HuN/42443/2015)^22^, as shown in Table 2, highlighting the potential ability of these new IAV-S reassortants to infect humans.

**Table 2:** Genome similarity of H1N1 novel reassortant viruses compared with the human isolated A/Hunan/42443/2015(H1N1)

Genotype	Name of virus	Gene segment (%)^a^							
		HA	NA	PB2	PB1	PA	NP	M	NS
C	A/swine/Guangxi/NN1994/2013	98.7	97.8	98.0	99.1	98.4	98.9	95.0	97.9
C	A/swine/Guangxi/CZ7/2014	98.0	98.0	97.9	99.0	97.8	98.7	92.8	97.7
C	A/swine/Guangxi/LB9/2014	98.0	97.9	97.9	99.0	97.8	98.7	94.8	97.6
C	A/swine/Guangxi/BS30/2014	98.0	98.0	97.9	98.9	97.8	98.6	94.8	97.7
C	A/swine/Guangxi /NNLX/2014	98.6	97.7	98.0	98.6	98.5	98.6	94.8	97.9
D	A/swine/Guangxi NNXD2023/2013	99.0	98.9	98.8	99.3	98.7	99.1	99.6	98.6
D	A/swine/Guangxi /QZ5/2014	98.6	98.1	97.9	99.0	98.5	98.6	97.3	98.0
D	A/swine/Guangxi /LZA3/2015	98.8	97.9	98.0	98.5	98.3	98.7	99.4	97.6

^a^The genome similarity was generated based on the open reading frame (ORF) sequences

### Molecular features

We next analyzed if these IAVs-S possessed key molecular features associated with receptor-binding ability, increased virulence, transmission, and antiviral resistance (Table 3).

**Table 3:** Amino acid substitutions in the novel H1N1 and H3N2 reassortant isolates compared with human isolates

Lineage/subtype	Host	Virus	No. of glycosylation sites in HA	HA^a^					PB2					M2	NA^c^
				138	190	225	226	228	271	590	591	627	701	31	274
EA H1N1	Human	A/Hunan/42443/2015	7	A	D	E	Q	G	A	G	R	E	D	N	Y
EA H1N1	Swine^b^	Sw/GX/NNXD2023/2013	6	*	*	*	*	*	*	S	*	*	*	*	*
EA H1N1	Swine^b^	Sw/GX/QZ5/2014	6	*	*	*	*	*	*	S	*	*	*	*	*
EA H1N1	Swine^b^	Sw/GX/LZA3/2015	6	*	*	*	*	*	*	N	*	*	*	*	*
EA H1N1	Swine^b^	Sw/GX/NN1994/2013	7	*	*	*	*	*	*	S	*	*	*	*	*
EA H1N1	Swine^b^	Sw/GX/CZ7/2014	6	*	*	*	*	*	*	S	*	*	*	*	*
EA H1N1	Swine^b^	Sw/GX/LB9/2014	6	*	*	*	*	*	*	S	*	*	*	*	*
EA H1N1	Swine^b^	Sw/GX/BS30/2014	6	*	*	*	*	*	*	S	*	*	*	*	*
EA H1N1	Swine^b^	Sw/GX/NNLX/2014	6	*	*	*	*	*	*	S	*	*	*	*	*
EA H1N1	Swine^b^	Sw/GX/BB2/2013	6	*	*	*	*	*	T	*	Q	*	N	*	*
EA H1N1	Swine^b^	Sw/GX/GG6/2013	6	*	*	*	*	*	T	*	Q	*	N	*	*
Early H1N1	Avian	A/duck/Schleswig/21/1979	6	*	E	G	*	*	T	*	Q	*	*	S	*
Pandemic H1N1	Swine^b^	Sw/GX/DX24/2013	6	*	*	D	*	*	*	S	*	*	*	*	*
Pandemic H1N1	Human	A/California/04/2009	6	*	*	D	*	*	*	S	*	*	*	*	*
HL H3N2	Swine^b^	Sw/GX/NNXD/2013	6	S	*	D	I	S	*	S	*	*	*	*	H
HL H3N2	Swine^b^	Sw/GX/JGB4/2013	6	S	*	D	I	S	*	S	*	*	*	*	H
HL H3N2	Swine^b^	Sw/GX/JG1/2014	6	S	*	D	I	S	*	S	*	*	*	*	H

*indicated the identical amino acids with A/Hunan/42443/2015; Sw represents swine, GX represents Guangxi

^a^H3 numbering

^b^isolates in this study

^c^N2 numbering

It is generally accepted that receptor-binding preference to human-type receptor is the initial key step for a novel influenza-virus-causing-pandemic. For H1 HAs, 190D and 225D/E are known to allow efficient binding to human-type α2–6 sialic acid linked receptors (H3 numbering, which is used throughout this work)^31,32^. All the EA H1N1 IAVs-S were highly conserved in the HA receptor-binding site, containing 138A, 190D, 225E, and 226Q, consistent with binding to α2–6 linked sialic acid. The H1N1/2009 pandemic-like swine isolate, Sw/GX/DX24/2013 contains 138A, 190D, 225D, and 226Q, also consistent with binding to α2–6 linked sialic acid. The α2–6 linked sialic acid receptor-binding specificity of H3 HAs is known to be determined by leucine and serine at positions 226 and 228. Our HL H3N2 viruses possessed characteristic residues found in human-adapted seasonal H3N2 viruses, such as 190D, 226I, and 228S^32^.

The presence of multiple basic amino acids at the HA cleavage site is characteristic of highly pathogenic influenza virus. All of our IAVs-S possessed a single basic amino acid (PSIQSR↓G or PEKQTR↓G) in the HA cleavage site. In addition, HA protein glycosylation is known to vary during influenza virus evolution^33^. One of the swine H1N1 isolates, Sw/GX/NN1994/2013, has seven HA potential glycosylation sites (Asn-X-Ser/Thr), which is the same number as the EA human strain (HuN/42443/2015). The remaining 13 IAVs-S had six HA potential glycosylation sites.

Among influenza virus proteins, PB2 is considered a major viral determinant of influenza viruses in mammals. The 627K and 701N amino acid residues in PB2 are known to play an important role in mammalian fitness for influenza viruses^34–38^. Most of IAVs-S with EA H1N1-origin PB2 genes have 701N. For H1N1/2009 pandemic virus, it was reported that 271A in PB2 enhances the transmissibility of influenza A in ferrets and 591R is important for H1N1/2009 pandemic in mammalian adaptation^38–40^. In our study, the IAVs-S with H1N1/2009 pandemic PB2 contain 271A and 591R.

Antiviral compounds are the first line of defense against novel influenza viruses until vaccines become available. Currently, two classes of drugs, adamantanes and NA inhibitors are available for treatment of influenza infections. The molecular marker of amantadine resistance, 31N in the transmembrane region of the M2 protein, is observed in all the isolates (Table 3)^41^. Meanwhile, H274Y mutation conferring resistance to NA inhibitors is also found in all the H1N1 swine isolates. However, the H3N2 IAVs-S do not have any known resistant mutation in the N2 against NA inhibitor.

### Seroprevalence of IAV-S in pigs of Guangxi

Of the 1170 serum samples collected from different cities in Guangxi, 170 (14.6%) were positive solely against EA H1, while 219 (18.8%) of the sera were positive solely for HL H3 swine virus (Table 4). Additionally, 126 (10.8%) were reactive against EA H1 and HL H3 according to the HI assay, indicating the possibility of a high frequency of coinfection, successive infection over time, and/or multiple infections by different IAV-S subtypes in the pig population. We did not test serologically for antibodies against other influenza A virus strains, our data are conservative estimates of the seroprevalence of IAV-S in the pig population.

**Table 4:** Seroprevalence of antibodies against different swine influenza virus in pigs in Guangxi from 2009 to 2013

Location	Serum samples^a^	No. (%) of sera positive		
		EA H1N1	HL H3N2	EA H1N1 + HL H3N2
Nanning	283	86 (30.4)	166 (58.7)	45(15.9)
Beihai	60	24 (40.0)	29 (48.3)	8(13.3)
Fangchenggang	40	3 (7.5)	5 (12.5)	1(2.5)
Liuzhou	101	28 (27.7)	17 (16.6)	8(7.9)
Laibin	130	31 (23.8)	4 (3.1)	2(1.5)
Guilin	135	33 (24.4)	33 (24.4)	16(11.9)
Hezhou	67	18 (26.9)	17 (25.4)	9(13.4)
Hechi	80	9 (11.2)	3 (1.3)	1(1.3)
Baise	60	6 (10.0)	1 (1.7)	0(0)
Yulin	134	44 (32.8)	51 (38.1)	25(18.7)
Guigang	80	14 (17.5)	19 (23.8)	11(13.8)
Total	1170	296 (25.3)	345 (29.5)	126(10.8)

^a^Eurasian avian-like (EA) H1N1, A/swine/Guangxi/BB2/2013(H1N1); Human-like swine (HL) H3N2, A/swine/Guangxi/JGB4/2013 (H3N2)

### Replication and virulence in mice

**Fig. 3:**
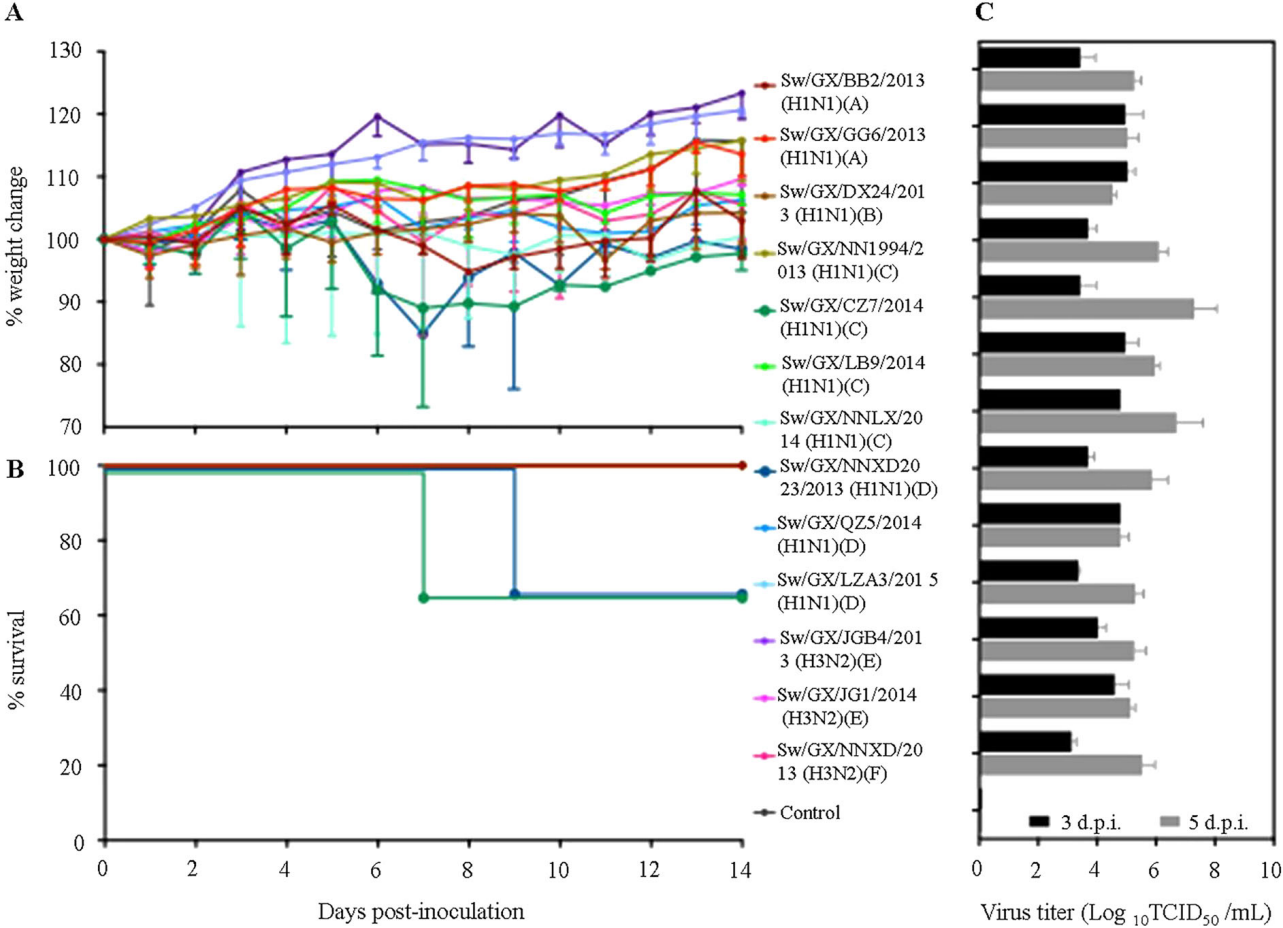
Weight variation (**a**), survival rates (**b**), and replication (**c**) of novel reassortant IAVs-S in mice. Mice in each group were infected intranasally with 5 × 10^4^ TCID_50_ of virus in a volume of 50 µL. The body weight and survival rates of mice were measured over 14 days. Virus titers of lung on 3 and 5 days post infection (d.p.i.) were shown as the mean titers of three mice

We selected ten H1N1 and three H3N2 influenza viruses, which included examples of each genotype and evaluated their replication and virulence in BALB/c mice. Groups of nine 6-week-old BABL/c mice were inoculated i.n. with 5 × 10^4^ TCID_50_ of virus in a volume of 50 µL. The average body weight loss of mice caused by two H1N1 viruses (Sw/GX/CZ7/2014 (genotype C) and Sw/GX/NNXD2023/2013 (genotype D)) reached to 15.5% and 15.1%, respectively (Fig. 3a), especially Sw/GX/CZ7/2014 caused 33% of mice death (Fig. 3b). The rest did not show obvious weight lost or death during the 2-week observation. All 13 viruses replicated in lungs of mice with titers ranging from 3.1 to 5.0 log_10_ TCID_50_/mL and 4.5 to 7.3 log_10_ TCID_50_/ml at days 3 and 5 p.i., respectively (Fig. 3c), with higher titers observed by the lethal Sw/GX/CZ7/2014 virus. There was no detection in spleens, kidneys, intestines, or brains of any mice. These results indicated that novel triple-reassortant viruses could replicate well in mice lung without prior adaptation and two H1N1 IAVs-S causing sign of disease and lethality. Interestingly, although it is known that H3N2 human viruses replicate to low levels in mice without adaptation^42^, this was not the case for the IAV-S H3N2 viruses isolated in our surveillance.

## Discussion

Here our surveillance study reveals that EA H1N1, H1N1/2009 pandemic, and HL H3N2 IAVs-S have been co-circulating in pigs in Guangxi, China from 2013 to 2015 (Table 1). Serological data suggested that multiple subtype exposure occurred (Table 4). Co-circulation of parent viruses in pigs could facilitate reassortment events. After the repeated introduction of pandemic H1N1 virus into pig population, novel reassortant viruses with H1N1/2009 pandemic-origin genes have been isolated frequently from pigs globally^19,43–45^. Notably, in this study, the predominant strains are novel triple EA H1N1 and HL H3N2 reassortants, which contains the CS H1N1 NS genes and the remaining five or four genes originating from H1N1/2009 pandemic. Swine triple-reassortant viruses with H1N1/2009 pandemic internal genes and CS H1N1 NS gene may have then become established in Southern China.

The transition from avian-type to human-type receptor-binding preference is a crucial step for influenza viruses to replicate efficiently in humans^46,47^. However, the specific amino acids that determine receptor-binding specificity vary among the different HA subtypes. For the H1 virus subtype, substitutions at E190D and G225D/E are important for the change in preference from avian to human receptors^31,48^. Yang et al.^25^ showed that pandemic H1N1 and EA H1N1 IAVs-S preferentially bind to human-type receptor. For the H3 virus subtype, substitutions Q226L and G228S are important for the change in preference from avian to human receptors^31,48^. Swine H3N2 displayed binding preference for α2, 6-SA receptors, if they possess the 190D, 226V/I/L, and 228S^32^. Our H1N1/2009 pandemic and all EA H1N1 IAVs-S possess 190D and 225D/E, and HL H3N2 IAVs-S contain 190D, 226I, and 228S, which indicates that these isolates possibly bind to human-type receptors with high affinity.

In recent years, concerns about low-pathogenic influenza has been increased, since it may be able to replicate well and possibly cause disease in humans, as observed during the 2009 H1N1 pandemic^4,49,50^. Mutations in the polymerase complex are critical for influenza viruses to infect and adapt mammals; notable examples of such mutations are 271A, 627K, 701N, and 591R^36–39,51,52^. Our prototypical EA H1N1 IAVs-S have 701N. For H1N1/2009 pandemic PB2, it is reported that 271T and 591R confer efficient replication and transmission in mammals^38,39^. All of our strains with H1N1/2009 pandemic PB2 bear 271A and 591R. These indicate that 14 IAVs-S in our study have mammalian-adapting mutations in their PB2 genes (Table 3). Importantly, IAVs-S containing these particular marker combinations are transmissible in ferrets, as previously described^25^.

A key prerequisite for influenza pandemic is that the virus becomes highly transmissible in humans. Numerous studies have reported that the internal genes from H1N1/2009 pandemic are a critical factor to promote aerosol transmissibility for reassortant viruses^38,39,50,52–56^. In this study, we identified novel swine triple H1N1 and H3N2 reassortants with similar internal genetic composition (genotypes C, D, and E). This provides direct evidence that pandemic H1N1 internal genes with or without the CS H1N1 NS gene complex have been successfully incorporated by reassortment and fixed into EA H1N1 and HL H3N2 IAVs-S at least during our surveillance period of time.

Currently, influenza A H1N1 and H3N2 viruses are the circulating seasonal influenza A viruses subtypes in human. The H1N1/2009 pandemic became the current seasonal H1N1 virus. Our EA H1N1 HAs share <73.7 and 78.1% similarity with the H1N1/2009 pandemic vaccine strain (A/Michigan/45/2015 H1N1), at nucleotide level and amino acid level, respectively. Our H3N2 IAVs-S share <94.1 and 91.5% similarity with the H3N2 vaccine strain (A/Hong Kong/4801/2014 H3N2), at nucleotide level and amino acid level, respectively. Studies have reported that seasonal trivalent inactivated influenza vaccine induce poor cross-reactive antibodies to EA H1N1 virus^23^ and does not protect against swine H3N2^57^. Importantly, according to the risk assessment tool, which is developed by the Centers for Disease Control and Prevention in the United States to evaluate the pandemic potential of different influenza strains^58^, we found that the EA H1N1 and swine H3N2 viruses are among the animal viruses with the highest risk score in Yang’s analysis^26^. Besides, at least one human infection with a similar reassortant IAV-S has been reported^22^. We suggest that intensive surveillance of IAV-S and of swine-to-human infections with the IAV-S described in our study should be a priority for future research.

## Electronic supplementary material

Figure S1 Phylogenetic analysis of the PB2 (A), PB1 (B), PA (C) and NP (D) of the 14 IAVs-S. The unrooted trees were based on nucleotides sequences of PB2, PB1, PA and NP and were generated with the MEGA 7.0 program by using neighbor-joining analysis and reliability of the tree was assessed by bootstrap analysis with 1000 replications. Neighbor-joining bootstrap values ≥70 are shown at the major branches of the trees
