# Supplementary figures and images for: Novel triple-reassortant influenza viruses in pigs, Guangxi, China
Source: Emerg Microbes Infect. 2018 May 16;7:85. doi: 10.1038/s41426-018-0088-z (PMC5953969; doi:10.1038/s41426-018-0088-z)

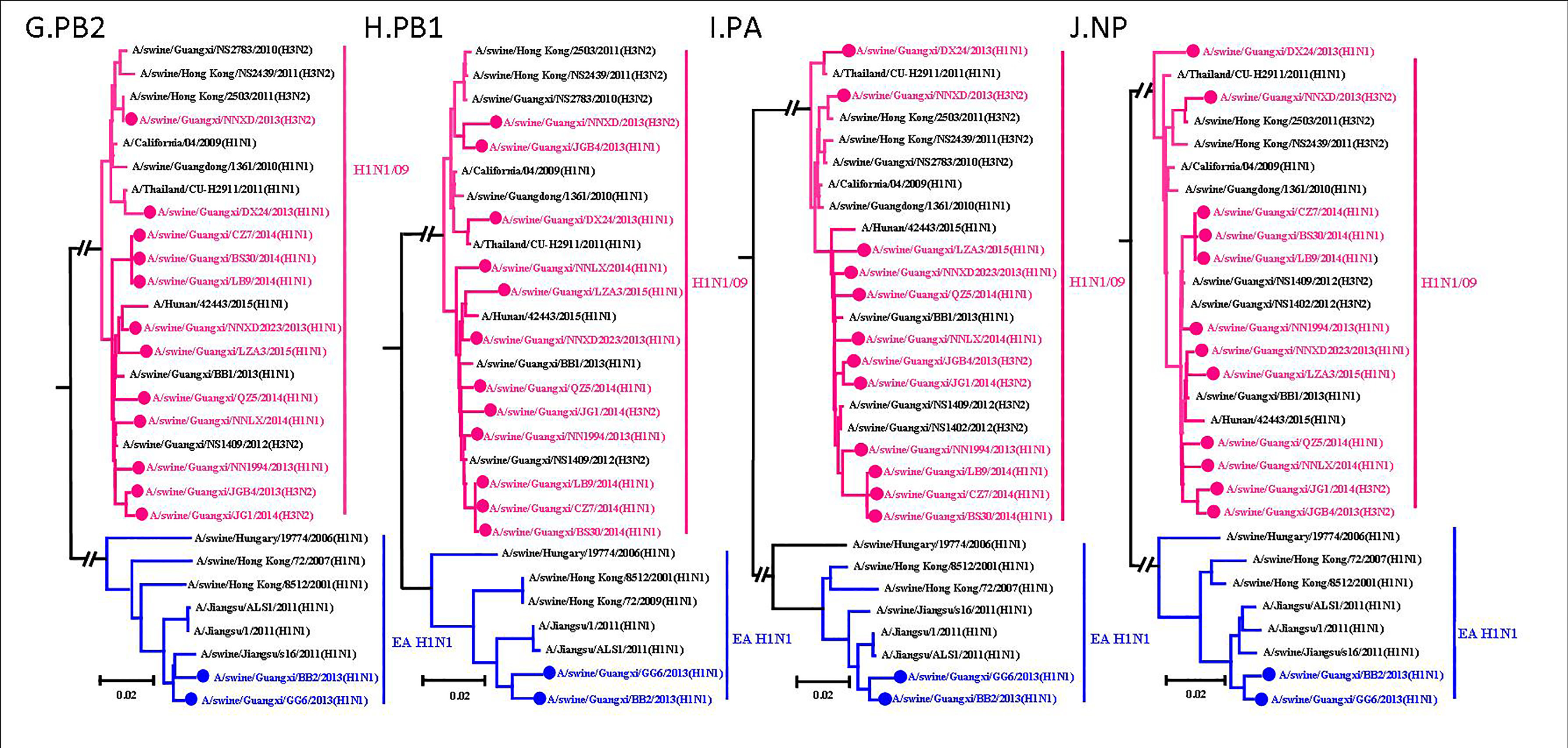

Supplement: Supplementary file 1 — Figure S1 Phylogenetic analysis of the PB2 (A), PB1 (B), PA (C) and NP (D) of the 14 IAVs-S. The unrooted trees were based on nucleotides sequences of PB2, PB1, PA and NP and were generated with the MEGA 7.0 program by using neighbor-joining analysis and reliability of the tree was assessed by bootstrap analysis with 1000 replications. Neighbor-joining bootstrap values ≥70 are shown at the major branches of the trees [file 41426_2018_88_MOESM1_ESM.jpg]
